# *M. leprae *inhibits apoptosis in THP-1 cells by downregulation of Bad and Bak and upregulation of Mcl-1 gene expression

**DOI:** 10.1186/1471-2180-6-78

**Published:** 2006-09-18

**Authors:** Zahra Hasan, Mussarat Ashraf, Ali Tayyebi, Rabia Hussain

**Affiliations:** 1 Department of Pathology and Microbiology, The Aga Khan University, Karachi, Pakistan

## Abstract

**Background:**

Virulent *Mycobacterium leprae *interfere with host defense mechanisms such as cytokine activation and apoptosis. The mitochondrial pathway of apoptosis is regulated by the Bcl-2 family of proteins. Expression of Fas ligand and apoptotic proteins is found in leprosy lesions and *M. leprae *has been shown to activate pro-apoptotic Bcl-2 genes, Bak and Bax. However, the mechanism by which *M. leprae *modulates apoptosis is as yet unclear. We investigated expression of apoptotic genes in THP-1 monocytes in response to infection by *M. leprae *and non-pathogenic *M. bovis *BCG.

**Results:**

*M. leprae *did not induce apoptosis in THP-1 cells, while BCG induced a significant loss of cell viability by 18 h post-infection at both (multiplicity of infection) MOI-10 and 20, with an increase by 48 h. BCG-induced cell death was accompanied by characteristic apoptotic DNA laddering in cells. Non-viable BCG had a limited effect on host cell death suggesting that BCG-induced apoptosis was a function of mycobacterial viability. *M. leprae *also activated lower levels of TNF-alpha secretion and TNF-alpha mRNA expression than BCG. *Mycobacterium*-induced activation of apoptotic gene expression was determined over a time course of infection. *M. leprae *reduced Bad and Bak mRNA expression by 18 h post-stimulation, with a further decrease at 48 h. Outcome of cell viability is determined by the ratio between pro- and anti-apoptotic proteins present in the cell. *M. leprae *infection resulted in downregulation of gene expression ratios, Bad/Bcl-2 mRNA by 39% and Bak/Bcl-2 mRNA by 23%. In contrast, live BCG increased Bad/Bcl-2 mRNA (29 %) but had a negligible effect on Bak/Bcl-2 mRNA. Heat killed BCG induced only a negligible (1–4 %) change in mRNA expression of either Bak/Bcl-2 or Bad/Bcl-2. Additionally, *M. leprae *upregulated the expression of anti-apoptotic gene Mcl-1 while, BCG downregulated Mcl-1 mRNA.

**Conclusion:**

This study proposes an association between mycobacterium-induced apoptosis in THP-1 cells and the regulation of Bcl-2 family of proteins. *M. leprae *restricts apoptosis in THP-1 cells by downregulation of Bad and Bak and upregulation of Mcl-1 mRNA expression.

## Background

Virulent mycobacteria such as *Mycobacterium leprae *and *M. tuberculosis *manipulate host cells to persist within them. Apoptosis, or programmed cell death is essential for the homeostatic regulation of cells, restriction of intracellular pathogens and also for stimulation of the host adaptive immune response. Virulent mycobacteria modulate host cell apoptosis to create a protected niche within cells [1-3], and downregulate protective host cytokines such as, TNFα and IFNγ [4,5] to reduce effector T cell and macrophage responses to the pathogen. Virulent *M. tuberculosis *and *M. bovis *induce lower levels of apoptosis than avirulent strains [6]. Virulent mycobacteria such as *M. leprae *induce reduced activation of pro-inflammatory cytokine such as TNFα, as compared with the non-pathogenic *M. bovis *BCG strain [7]. The apoptotic response to mycobacteria is found to be dependent on cytokine activation [8] whereby, TNFα has been shown to activate mycobacterium-induced apoptosis[9], while IL-10 downregulates apoptosis in macrophages [10].

*M. leprae *causes a disease spectrum ranging from disseminated multibacillary (MB) leprosy to restricted paucibacillary (PB) disease. T cell and macrophage responses are less affected in PB leprosy than in MB, where effector T cell and macrophage responses are severely compromised. Multibacillary leprosy granulomas show increased expression of Fas ligand which may protect these sites from attack by Fas-expression cytotoxic T lymphocytes [11]. Production of pro-inflammatory cytokines such as TNFα is found to be greater in PB disease as compared with MB disease [12]. Although apoptotic proteins are activated in leprosy lesions from all patients [13], reports indicate that apoptosis is greater in PB disease, correlating with increased TNFα secretion. A role for TNFα is further corroborated as *M. leprae *induced apoptosis is reduced by treatment with TNFα inhibitor pentoxyfylline [14] or anti-TNFα antibodies [15].

Regulation of programmed cell death occurs via either the intrinsic pathway, involving mitochondrial release of cytochrome C and activation of caspase 9, or the extrinsic pathway, involving the stimulation of death receptors expressed on the cell surface and activation of caspase 8. The Bcl 2-family of proteins comprise of anti-apoptotic members; Bcl-2, Bcl-x_L _and Mcl-1, and pro-apoptotic members; Bad, Bid, Bax and Bak. Pro-apoptotic Bcl-2 proteins located in the cytosol act as sensors of cellular stress and damage [16]. Upon activation, they relocate to the mitochondrial surface where anti-apoptotic proteins are located. Activation of Bax or Bad and blockade of anti-apoptotic Bcl-2 proteins are pivotal steps in the mitochondrial pathway of apoptosis. Bad positively regulates cell apoptosis by forming heterodimers with Bcl-x_L _and Bcl-2 and reversing their death repressor activity. Pro-apoptotic Bak is sequestered by Mcl-1 and Bcl-x_L_; but not Bcl-2 until displaced by Bid, Bad or Bak [17]. When there is an excess of pro apoptotic proteins, cells are more sensitive to apoptosis.

Mycobacteria interfere with host apoptosis by modifying expression of Bcl-2 proteins. Virulent *M. tuberculosis *has been shown to repress apoptosis by upregulation of Bcl-2 [18] and Mcl-1 [19], and by deactivation of Bad [20] and Bax [2]. Previously, *M. leprae *has been shown to induce apoptosis in monocytes via induction of Bak and Bax genes [14]. However, the mechanism by which *M. leprae *influences host cell apoptosis is as yet unclear.

The human acute monocytic leukemia cell line THP-1 has been established as a model to study mycobacterial growth and persistence [21] in addition to mycobacterium-induced apoptosis [22-24]. Here we have investigated the mechanism by which virulent *M. leprae *regulates apoptosis in THP-1 human monocytes as compared with the non-pathogenic *M. bovis *BCG vaccine strain.

## Results

### *Mycobacterium leprae *induces less cell death as compared with *M. bovis *BCG

**Figure 1:**
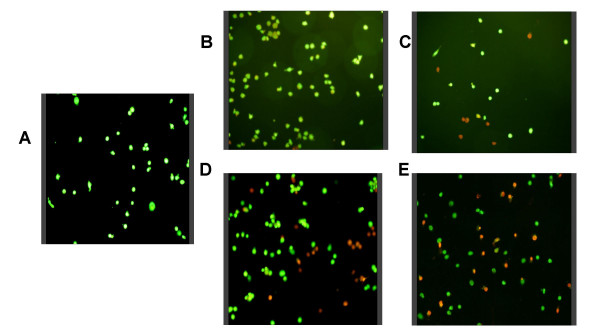
***Mycobacterium*-induced cell death in THP-1 cells**. THP-1 cells were plated on coverslips at 2 × 10^5 ^per well and infected with either *M. leprae *or BCG at a multiplicity of infection (MOI) of 10 and 20 per cell for 18 h. Cells were stained for viability using acridine orange – ethidium bromide under which, viable cells appear green while apoptotic cells appear red/orange. A. unstimulated cells, B – C. *M. leprae *MOI-10 and MOI-20, D – E. BCG MOI-10 and MOI-20.

**Figure 2:**
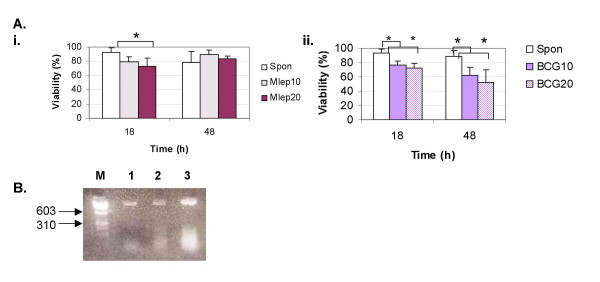
***M. leprae *induces a lower rate of cell death as compared with BCG**. THP-1 cells were infected with *M. leprae *and BCG as described in Fig. 1. A. Cellular viability was assessed after fluorescent staining at 18 and 48 h post-infection. Graphs show the results from 5 independent experiments with SD shown as 'y' error bar. '*' denotes significant differences (P < 0.05). (i) *M. leprae*, (ii) BCG. B. Agarose gel electrophoresis of internucleosomal DNA fragmentation in THP-1 cells shows spontaneous *(lane 1)*, or BCG-induced apoptosis at MOI-10 *(lane 2) *and MOI-20 *(lane 3) *after 18 h of culture. The molecular markers are indicated to the left *(lane M)*.

*M. leprae *and BCG-induced death in THP-1 cells was evaluated by fluorescent viability staining. A time course of infection was carried out at 6, 18 and 48 h post-stimulation. Viability was determined after infection with *Mycobacterium *at a multiplicity of infection (MOI) of 10 and 20 in each case. Neither *M. leprae *nor BCG induced any significant loss in cell viability at the earlier 6 h time interval studied (data not shown). After 18 h of culture *M. leprae *did not cause any significant loss of viability in THP-1 cells at MOI-10 however, cell death was evident in cells infected at MOI-20 (Figs. 1B–C. As shown in Fig. 2A, *M. leprae *induced significant cell death induction at MOI-20 (P < 0.01) at 18 h but not at either MOI 10 or MOI-20 (P = 0.052) by the later 48 h time interval. In contrast, BCG infection of THP-1 cells resulted in a significant increase in cell death at both MOIs 10 and 20 at 18 h (P < 0.01) (Fig. 1D–E) and this effect was also evident at 48 h (P < 0.01) (Fig. 2.A2).

The pattern of genomic DNA fragmentation in mycobacterium infected cells was determined in order to investigate the cause of loss in cell viability observed at 18 h post-infection. BCG-infection of cells resulted in DNA laddering characteristic of apoptotic cells, with an increase in apoptosis with dose from MOI-10 to MOI-20 (Fig. 2B). In contrast, apoptotic laddering was not observed in cells infected with *M. leprae *(data not shown).

We employed gamma-irradiated *M. leprae *in our assays as this was the only strain available to us and we have previously used it to model *M. leprae *infections in cells from both healthy controls and leprosy patients [25,26]. The *M. bovis *BCG strain served as a comparison of a non-pathogenic species, while heat-killed BCG was employed as a control for BCG viability in the assays. Heat-killed BCG at the lower infection dose of MOI-10 did not cause any significant loss in THP-1 viability but did so at MOI-20 (P < 0.05) at 18 h. However, cell death was not induced by heat-killed BCG at 48 h post-infection (data not shown).

### *M. leprae *is a poorer inducer of TNFα than *M. bovis *BCG

**Figure 3:**
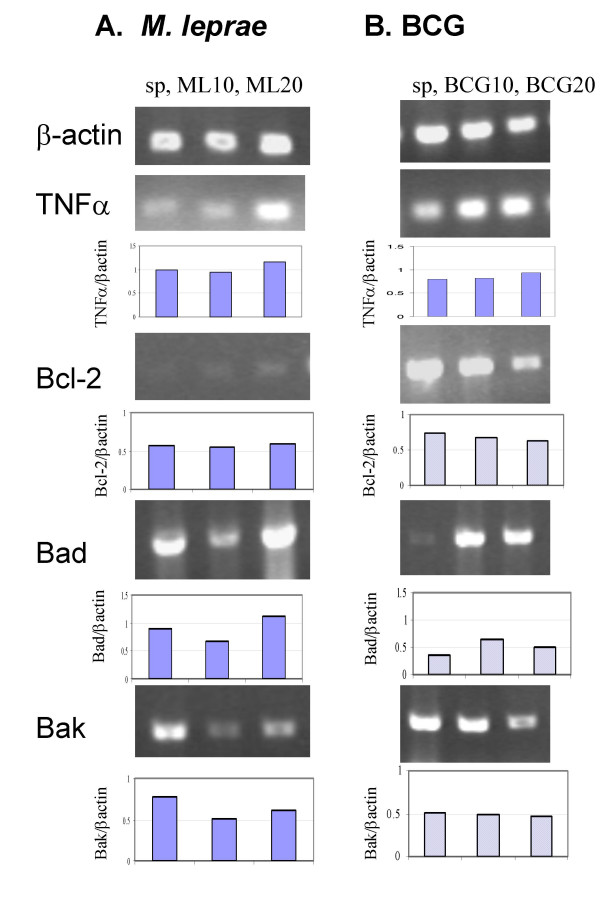
***Mycobacterium*-induced TNFα, Bcl-2, Bad and Bak gene expression**. THP-1 cells were infected with *M. leprae *and BCG as described previously and total cellular RNA was extracted, reverse transcribed and PCR was carried out for β-actin, TNFα, Bcl-2, Bad and Bak genes. Gene expression was quantified using scanning densitometry. Panels illustrate gene expression in unstimulated cells 'sp', *M. leprae *'ML10', 'ML20', and BCG 'BCG10' and 'BCG20' infected cells at 18 h post-stimulation in a representative experiment. Graphs illustrate expression of TNFα, Bcl-2, Bad and Bak mRNA relative to β-actin in each case.

A role for TNFα is indicated in mycobacterium-induced apoptosis. *M. leprae*-induced apoptosis in primary macrophages has been shown to be dependent on TNFα activation [14], while BCG-induced apoptosis in THP-1 cells is dependent on an endogenous TNF response [24]. Virulent mycobacteria are poorer inducers of TNFα than avirulent strains [27,28]. *M. leprae *has previously been shown to induce only negligible TNFα secretion in THP-1 cells [29]. We investigated *M. leprae *induced TNFα secretion and mRNA expression in THP-1 cells to determine whether the low level of apoptosis observed could be associated with a lack of TNFα activation in the assay. *M. leprae *and BCG -induced cytokine secretion was measured at 6, 18 and 48 h post-stimulation. BCG infection of cells induced TNFα secretion within 6 h of stimulation (mean values: BCG10, 40 pg/ml; BCG20, 80 pg/ml) with peak secretion at 18 h (mean values: BCG10, 238 pg/ml; BCG20, 299 pg/ml) and a decrease at 48 h (mean values: BCG10, 165 pg/ml; BCG20, 204 pg/ml). *M. leprae *did not induce any detectable TNFα secretion in THP-1 cells at either MOI 10 or 20 at 6 h, with negligible secretion (ML10, 0 pg/ml; ML20, 4 pg/ml) at the 18 h peak interval. However, *M. leprae *induced TNFα mRNA expression upon infection of cells but at a lower level than as compared with BCG (Fig 3).

### Bak and Bad gene expression is down-regulated in *M. leprae *infected THP-1 cells

**Figure 4:**
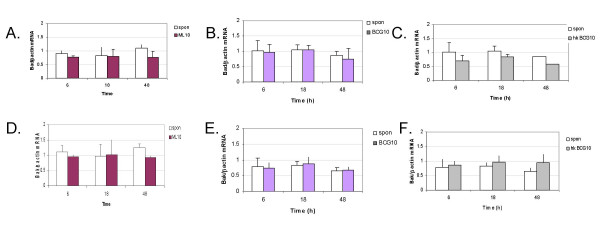
***M. leprae*-, and BCG- induced time course expression of Bad and genes**. THP-1 cells were infected with *M. leprae*, live and heat-killed BCG for 6, 18 and 48 h at MOI of 10 each. RNA was extracted, converted to cDNA and PCR carried out for Bad, Bak and β-actin genes. Graphs illustrate results obtained from the mean of 3 independent experiments. A, D. *M. leprae*-, B, E. live BCG- and C, F. heat killed BCG-induced Bad/βactin and Bak/βactin mRNA expression ratios; Spon (unstimulated cells).

*M. leprae *infection of peripheral blood monocytes has been shown to result in the activation of the pro-apoptotic protein Bad and induction of Bak mRNA [14]. In this study we did not observe apoptosis in THP-1 cells infected with *M. leprae*, while cells infected with BCG showed increased apoptosis. To investigate the mechanism responsible for this difference we measured *Mycobacterium*-induced mRNA expression of pro-apoptotic (Bad and Bak) and anti-apoptotic (Bcl-2) genes at 6, 18 and 48 h post infection. Gene expression was compared relative to that of the β-actin housekeeping gene in each case. Fig. 3 illustrates the changes in gene expression observed in response to *M. leprae *and BCG infection of cells at MOIs of 10 and 20. *M. leprae *induced negligible effect on Bcl-2 mRNA at both infection doses, while Bad mRNA expression appeared to be reduced at MOI-10 but increased at MOI-20 (Fig. 3A). *M. leprae *downregulated Bak expression at both infection doses studied. In contrast, BCG infection of monocytes downregulated Bcl-2 mRNA but upregulated Bad mRNA expression at both MOIs 10 and 20, while it had negligible effect on Bak mRNA expression (Fig. 3B). The data shown is of one representative experiment and some variation is evident between background levels of mRNA expression in different experiments. To account for this, in each case mRNA levels for each gene were normalized to baseline levels of β-actin within the same experiment. Further time course kinetics of apoptotic mRNA expression based on data from 3 independent experiments is presented in Fig. 4. The effect of heat-killed BCG on THP-1 cells is also included. Negligible change was observed in Bad or Bak gene expression in response to *M. leprae *or BCG (live and heat-killed) at the earlier 6 and 18 h time intervals. However by 48 h, *M. leprae *induced downregulation of mRNA expression both pro-apoptotic genes Bad (30 %) and Bak (26 %) (Fig. 4A, D). In contrast, live BCG induced negligible change in either Bad or Bak mRNA expression at 48 h post-infection (Fig. 4B, E). Killed BCG induced downregulation of Bad mRNA (34 %) but upregulation of Bak mRNA (46 %) at 48 h post-infection (Fig. 4C, F).

**Figure 5:**
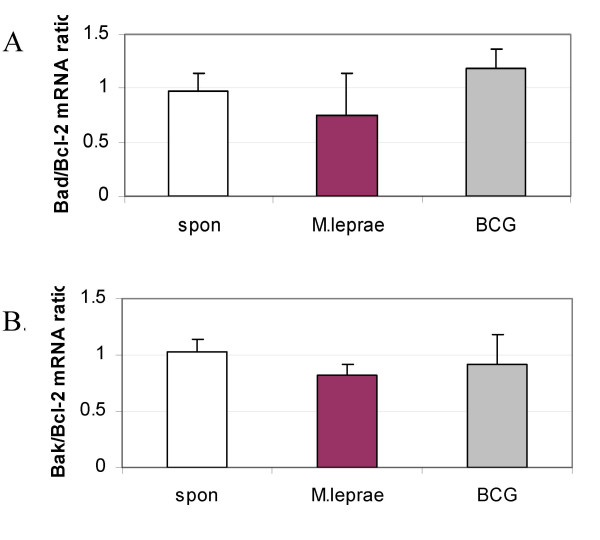
**Differential downregulation of Bad/Bcl-2 and Bak/Bcl-2 mRNA by *M. leprae *as compared with BCG**. Gene expression was determined in cells infected with either *M. leprae *or BCG at 18 h post-infection. mRNA expression was quantified using densitometry and graphs illustrate the mean pro-/anti-apoptotic gene ratios of 3 independent experiments. A. Bad/Bcl-2 and B. Bak/Bcl-2 mRNA expression.

Outcome of cellular viability/apoptosis is determined not only by the expression of specific pro- and anti-apoptotic genes, but is dependent on the ratio between pro and anti apoptotic genes. Therefore, to further understand *Mycobacterium*-induced change in host apoptotic gene expression we determined Bad/Bcl-2 and Bak/Bcl-2 expression ratios. We chose to evaluate gene expression at 18 h post-infection, the time interval at which differential cell death induction is evident between *M. leprae *and BCG (MOI-10 (Fig. 1). *M. leprae *infection of THP-1 cells resulted in a 39 % decrease in Bad/Bcl-2 and a 23 % reduction in Bak/Bcl-2 gene expression as compared with unstimulated cells (Fig. 5A–B) while *M. leprae *at MOI 20 caused a 54 % decrease in Bad/Bcl-2 and 15 % decrease in Bak/Bcl-2 mRNA expression (data not shown). BCG infection of cells at MOI-10 increased Bad/Bcl-2 by 29 % and reduced Bak/Bcl-2 mRNA expression by 4 %, with a further increase in Bad/Bcl-2 mRNA at MOI-20 and similar change in Bak/Bcl-2 mRNA. Killed BCG had a limited effect on Bad/Bcl-2 (1–13 %) and Bak/Bcl-2 (4–6 %) mRNA expression at MOIs 10 and 20 (data not shown).

### *M. leprae *upregulates Mcl-1 mRNA expression

**Figure 6:**
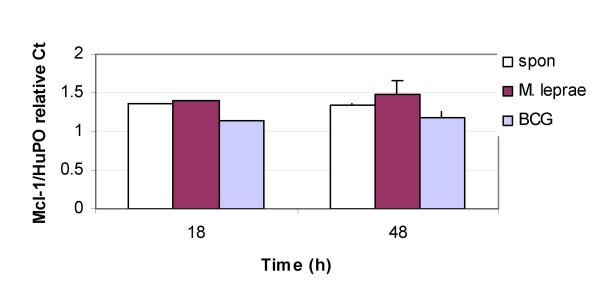
***M. leprae *upreglates Mcl-1 mRNA in THP-1 cells**. THP-1 monocytes were infected with *M. leprae *or BCG at MOI-10 and RNA was harvested after 18 and 48 h of culture. mRNA was reverse transcribed and used for real-time PCR quantification of Mcl-1 and HuPO genes. Relative quantification data of 3 independent experiments calculated using the C_T _method is illustrated.

Survival of *M. tuberculosis *infection in THP-1 cells has been linked to the upregulation of anti-apoptotic Bcl-2 family member Mcl-1 [19]. However, a role for Mcl-1 in *M. leprae *or BCG infection of cells is not known. Therefore, we investigated *M. leprae *and BCG induced Mcl-1 gene expression in THP-1 cells. A time course of *Mycobacterium-*induced Mcl-1 expression was determined at 6, 18 and 48h. Mcl-1 was evaluated relative to that of the housekeeping gene human ribosomal protein (HuPO), which has been shown to be optimal for real-time PCR quantification of gene expression in human cells [30]. At the earlier 6 h time point neither *M. leprae *nor BCG infection of THP-1 cells had any effect on Mcl-1 mRNA expression as compared with unstimulated cells (data not shown). *M. leprae *induced an increase in Mcl-1 expression by 2.9 % at 18 h post-infection with a further (12 %) increase at 48 h. In contrast, BCG induced a decrease in Mcl-1 mRNA by 15 % at 18 h and 12 % by 48 h (Fig. 6).

## Discussion

Our study shows that *M. leprae *inhibits apoptosis in the THP-1 monocytic cell line by downregulation of proapoptotic genes Bak and Bad and concomitant upregulation of anti-apoptotic Mcl-1 mRNA. In contrast, the increased apoptosis evidenced by BCG infection of THP-1 cells is coordinated by an increase in Bad mRNA expression relative to Bcl-2 and a downregulation of Mcl-1 mRNA.

Our data correlates with previous reports that virulent mycobacteria induce lower levels of cell death than avirulent mycobacteria [6]. We did not observe *M. leprae-*induced loss in cell viability in cells infected at MOI-10 at 18 post-infection. While, some THP-1 cell death was caused by *M. leprae *at MOI 20 by 18 h post-infection, this was not accompanied by the characteristic DNA laddering pattern found in apoptotic cells. Therefore, it is possible that the loss of viability observed here could be due to an alternate mechanism of cell death such as, necrosis, and could also be attributed to the increased mycobacterial load in the THP-1 cells. However, at the later time interval of 48 h *M. leprae *-induced cell death was not observed at either infection dose. This may be attributed to the time required for *M. leprae *-induced modification of host gene expression which facilitates persistence of the pathogen within cells. This is further evidenced by the time course changes in mRNA expression of the Bcl-2 family of proteins observed in this study.

Cell death induced by BCG was dose dependent as shown previously [24]. BCG-induced apoptosis (as shown by DNA laddering of cells) increased with time from 18 to 48 h post-infection. Loss in cell viability was observed in response to heat-killed BCG at 18 h but was no longer apparent by 48 h. The earlier response to non-viable BCG observed at 18 h may be the result of non-specific binding and entry of the mycobacterium into the host cell which may trigger host defenses. Initial cell attachment and uptake of BCG into host macrophages has shown to be common amongst live and dead mycobacterium, with subsequent differences occurring later due to differential trafficking of live and dead organisms within host the endosomal/lysosomal pathway [31]. As BCG-induced apoptosis is most probably dependent on bacterial viability it is therefore only sustained in the case of live BCG.

*M. leprae-*induced less TNFα secretion and gene expression than BCG did. A role for TNFα has been defined previously in BCG-induced apoptosis in THP-1 cells [24]. In addition, *M. leprae*-induced apoptosis in primary monocytes has been shown to be TNFα dependent [14], and inhibition of TNFα via treatment with anti-TNFα inhibitors or pentoxyfylline is found to reduce host inflammation and cell death in reactional leprosy [32]. Therefore, in the case of *M. leprae *infection, the reduced levels of TNFα activation coordinate with the downregulation of pro-apoptotic gene expression would contribute to apoptosis inhibition in the cells.

We found *M. leprae *to downregulate expression of pro-apoptotic genes Bak and Bad relative to both β-actin and anti-apoptotic Bcl-2. This correlates with previous studies where reduced Bax and increased Bcl-2 mRNA expression has been shown to be responsible for slowly progressive murine tuberculosis [33]. The role of Bad gene expression in determining outcome of cellular viability is further indicated where cell wall lipoarabinomannan (LAM) from *M. tuberculosis*-induces survival in monocytes by phosphorylation of the protein Bad, hence preventing it from binding Bcl-2 [22]. However, our work differs from that by Hernandez, *et *al. who demonstrate that infection of primary monocytes with irradiated *M. leprae *resulted in apoptosis due to activation of apoptotic genes, Bax and Bak [14]. This apparent discrepancy can be attributed to differential outcome of mycobacterial infection dependent on host cell type, as evidenced by the variation in apoptosis induction by *M. tuberculosis *observed in alveolar epithelial cells and U937 macrophages [20]. In addition, Hernandez et al [14] showed that *M. leprae *increases apoptosis in monocytes-derived macrophages of leprosy patients. However, macrophages from leprosy patients are more prone to apoptotic death than those from healthy individuals [15]**. **Therefore, the spontaneous activation of apoptotic genes in leprosy patients may make the cells more prone to extracellular triggers such as infection with mycobacteria.

We demonstrate a role for Mcl-1 in *M. leprae *mediated inhibition of apoptosis in the THP-1 cells. The anti-apoptotic Bcl-2 family member gene Mcl-1 has been shown to play a role in cell survival of human polymorphonuclear cells [34] and neutrophils [35]. Virulent *M. tuberculosis *H37Rv has been shown to persist in THP-1 cells by upregulation of the anti-apoptotic Bcl-2 family member gene Mcl-1 [19]. Pro-apoptotic Bak is sequestered by Mcl-1 [17] therefore upregulation of Mcl-1 expression would result in a coordinate decrease in available Bak leading to further downregulation of apoptosis.

We found BCG-induced apoptosis to be accompanied with increased expression of Bad/Bcl-2 mRNA. Previously, BCG-induced apoptosis has been shown to be a consequence of downregulated Bcl-2 expression [1] and also by activation of caspases [24]. We show for the first time in our study that BCG induces downregulation of anti-apoptotic Mcl-1 mRNA. Overall, our results indicate that the differential effect of virulent *M. leprae *and non-pathogenic BCG on host cellular survival is determined by the combined gene expression of pro- and anti-apoptotic genes Bad, Bak, Bcl-2 and Mcl-1.

## Conclusion

*M. leprae-*induced downregulation of Bad and Bak genes coordinate with a reduced TNFα response and upregulation of Mcl-1 results in the inhibition of apoptosis in THP-1 monocytes. Therefore, leading to a more efficient mechanism for dissemination of leprosy infection.

## Materials and methods

### Mycobacterial strains used

*M. bovis *BCG (Montreal vaccine strain) was used as described previously [7]. Gamma-irradiated *M. leprae *(prepared from armadillo liver tissue) was provided by Colorado State University, USA, by NIH contract (NO1-AI-55262, 'Leprosy research support'). BCG was were cultured in 7H9 Middlebrook medium supplemented with 0.02 % glycerol, 10 % ADC Middlebrook enrichment and 0.5 % Tween-80 (DIFCO Laboratories, Detroit, MI, USA). BCG was heat killed by treatment at 80°C for 30 min. *Mycobacterium *sp. was stored in single use aliquots at -70°C and used as described previously [25].

### *Mycobacterium *infection assays

The THP – 1 human macrophage-like cell line was acquired from the American Type Culture Collection, USA (TIB-202) and cultured in RPMI-1640 medium containing 2 mM L-glutamine, 10 mM HEPES, 1 mM sodium pyruvate, 4.5 g/L glucose, 1.5 g/L bicarbonate, supplemented with 10 % heat inactivated fetal calf serum and 0.05 mM β-mercaptoethanol. Monocytes were seeded at 2 × 10^5 ^cells/well in 24 well plates and differentiated by stimulation with 20 ng/ml phorbol myristate acetate (PMA) for 18–20 h. Cells were subsequently washed with sterile PBS and infected with either BCG or *M. leprae *at a dose of 10 or 20 bacteria per cell. Supernatants were harvested at 6, 18 and 48 h as per experiment and stored at -70°C until measured.

### ELISA for TNFα

ELISA reagents for TNFα were from R&D Systems (USA). Assays were carried out according to the manufacturer's recommendation and as reported previously. [7] The lower limit of detection was 3.9 pg/ml.

### Fluorescence viability staining

For fluorescence assays, cells were seeded on glass coverslips within 24 well plates as described above. Staining dye cocktail contained acridine orange (100 μg/ml) and ethidium bromide (100 μg/ml) in PBS [36]. One ml of dye cocktail was added to each coverslip, incubated in the dark for 2 min and subsequently visualized by microscopy.

### Apoptotic DNA laddering

THP-1 cells (2 × 10^5^/sample) were lysed, and the genomic DNA extracted according to the protocol for TACS DNA Laddering Kit (R&D Systems, USA). Samples were analyzed on a 1.5 % agarose gel with ethidium bromide staining.

### Gene expression quantification for βactin, Bcl-2, Bak and Bad

Total cellular RNA was harvested from adherent cells using Trizol reagent (GIBCO-BRL, USA). cDNA was prepared by reverse transcription using MuLV reverse transcriptase and PCR for the β-actin gene was carried out using custom designed primers as described previously [25]. PCR primers for Bcl-2, Bak and Bad were [14,20] F ;5' TGATTGAAGAGACCCCCTCGT 3'; Bcl-2-R: 5'GTCAGGTTGGGAGTGAACGCT 3'; Bak-F 5' GCCCAGGACACAGAGGAGGTTTTC-3'; Bak-R 5' AAACTGGCCCAACAGAACCACACC-3'; Bad-F 5'- GAGCCCGGGGTGCTGGAGGGA-3'; Bad-R 5'-GGCGGCACAGACGCGGGCTTT-3'. Typical PCR conditions were; 95°C, 1 min; 30 cycles, 95°C, 45 Sec, 60°C,.45s; 72°C, 1 min; 72°C,1 min; extension 72°C,10 min.

### Statistical analysis

The non-parametric Mann-Whitney U test was carried out for pairwise comparison of samples using SPSS software, USA.

### Gene expression quantification of Mcl-1 using real-time PCR

Gene expression was evaluated by relative quantification using the comparative threshold C_T _method. PCR primers for Mcl-1 were as described previously [19] and gene expression was expressed relative to that of house keeping gene, human ribosomal protein (HuPO) gene [30]. Thanks for HuPO primer sequences to Jackie Cliff. PCR was carried using the SYBR green master mix on the iCycler, BIORAD, USA.

## Abbreviations

multiplicity of infection (MOI), *M. leprae *(ML)

## Authors' contributions

This work was conceived, worked upon and written up by ZH. AT carried out culture and microscopy, while MA performed *Mycobacterium *infection assays and gene expression analysis. RH provided critical advice and helped in manuscript preparation. All authors read and approved the final manuscript.
